# An antimicrobial peptide specifically active against Listeria monocytogenes is secreted by Bacillus pumilus SF214

**DOI:** 10.1186/s12866-021-02422-9

**Published:** 2022-01-03

**Authors:** Anella Saggese, Ylenia De Luca, Loredana Baccigalupi, Ezio Ricca

**Affiliations:** 1 Department of Biology, Federico II University, via Cinthia, Complesso Universitario di Monte Sant’Angelo, 80126 Naples, Italy; 2 Department of Molecular Medicine and Medical Biotechnology, Federico II University, Naples, Italy

**Keywords:** *Bacillus*, Antimicrobials, AMP, anti-*Listeria*, Lytic peptide, Membrane damages

## Abstract

**Background:**

Members of the *Bacillus* genus produce a large variety of antimicrobial peptides including linear or cyclic lipopeptides and thiopeptides, that often have a broad spectrum of action against Gram-positive and Gram-negative bacteria. We have recently reported that SF214, a marine isolated strain of *Bacillus pumilus*, produces two different antimicrobials specifically active against either *Staphylococcus aureus* or *Listeria monocytogenes.* The anti-*Staphylococcus* molecule has been previously characterized as a pumilacidin, a nonribosomally synthesized lipopetide composed of a mixture of cyclic heptapeptides linked to fatty acids of variable length.

**Results:**

Our analysis on the anti-*Listeria* molecule of *B. pumilus* SF214 indicated that it is a peptide slightly smaller than 10 kDa, produced during the exponential phase of growth, stable at a wide range of pH conditions and resistant to various chemical treatments. The peptide showed a lytic activity against growing but not resting cells of *Listeria monocytogenes* and appeared extremely specific being inactive also against *L. innocua*, a close relative of *L. monocytogenes*.

**Conclusions:**

These findings indicate that the *B. pumilus* peptide is unusual with respect to other antimicrobials both for its time of synthesis and secretion and for its strict specificity against *L. monocytogenes*. Such specificity, together with its stability, propose this new antimicrobial as a tool for potential biotechnological applications in the fight against the dangerous food-borne pathogen *L. monocytogenes.*

## Background

Antibiotic resistance is a major threat to global health and is predicted to cause by 2050 millions of annual deaths [1]. In addition to an increased incidence of infectious diseases, the lack of effective antibiotics will certainly affect many surgical procedures and treatments that suppress the immune system (for example, chemotherapies) [1]. Therefore, the identification of new strategies to fight the continuous rise of resistant pathogens is an enormous challenge for the scientific community [1, 2]. So far, alternatives to antibiotics such as vaccines and probiotics, have been proposed together with the identification and design of new antibiotics.

In this context, antimicrobial peptides (AMPs) have gained great attention as new antibiotics. They have been characterized from almost every organism of all domains of life and shown to play an important role in innate immunity, protecting the producing organisms from infections [1]. Many AMPs act on membrane lipids and show a broad-spectrum of action against Gram-positive and Gram-negative bacteria, some AMPs are active against fungi, viruses or other parasites [1]. AMPs from vertebrates (defensins and cathelicidins), in addition to the antimicrobial activity, also act as chemo-attractants, are involved in the activation of complement pathways and are able to modulate the immune response [1]. Over 3,000 AMPs, showing an extraordinary chemical diversity and isolated from a variety of biological sources, have been described and are collected in specific databases (see for example: http://aps.unmc.edu/AP/main.php; https://wangapd3.com/main.php; https://dbaasp.org/).

Bacteria and fungi in particular have been investigated as a source of new AMPs, and although most microorganisms produce AMPs, bacteria of the *Bacillus* genus have received deep attention as potential starting points in the search for new inhibitory substances [2]. These rod-shaped, endospore-forming, Gram-positive bacteria are common in many natural habitats and produce a variety of AMPs: short linear or cyclic peptides of up to 30 amino acid residues; lipopeptides containing a hydrocarbon tail linked to the N-terminus of the linear or cyclic peptide; thiopeptides, sulphur-rich peptides with thiazole, oxazole, or thiazoline rings; and 2,5-diketopiperazines (DKPs), extremely small peptides (even di-amino acids) with structural modifications [2, 3].

The best known AMPs produced by Bacilli are cyclic lipopeptides of three structural categories: surfactins, iturins and fengycins. In addition, tens of linear or cyclic lipopeptides not belonging to those three classes, have been identified and extensively reviewed by Zhao et al. [2] and Cauller et al. [3].

In Bacilli AMPs biosynthesis principally occurs by two different biosynthetic pathways: the non-ribosomal synthesis of peptides by large mega-enzymes, the non-ribosomal peptide synthetases (NRPSs) [4, 5], and the ribosomal synthesis of linear precursor peptides that are subjected to post-translational modifications and proteolytic processing [6, 7].

We have previously reported that SF214, a marine isolated strain of *B. pumilus*, produced two independent antimicrobials specifically active against *Staphylococcus aureus* or *Listeria monocytogenes* [8]. SF214 is known to produce a water soluble yellow-orange pigment, essential to protect the bacterium against oxidative stresses [9, 10]. Synthesis of the still uncharacterized pigment of SF214 is a bistable process that occurs in alternative to spore formation [10]. Pigment synthesis, sporulation and matrix synthesis in SF214 are connected phenomena, controlled by the same master regulators [11]. The antimicrobial molecule active against *S. aureus* has been previously characterized as a pumilacidin, a nonribosomally synthesized cyclic lipopeptide of the surfactin class, composed of seven amino acid residues linked to fatty acids of variable length [8]. Here is reported that the anti-*Listeria* molecule produced by SF214 is a peptide with lytic and specific activity against growing cells of *Listeria monocytogenes* and that it is stable at conditions of low pH and high temperature.

## Results and Discussion

### Exponentially growing cells of ***B. pumilus*** SF214 produce a stable anti-***Listeria*** molecule

**Fig. 1:**
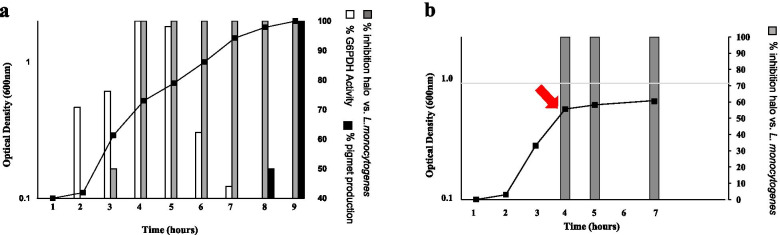
Production of the antimicrobial during growth (**a**) and after a treatment with chloramphenicol (**b**). In (**a**), aliquots of the cell culture were collected at all-time points, the cell-free supernatant size-fractionated and the >10 kDa fraction tested against *L. monocytogenes* [8]. For all time points, cell extracts were analyzed for G6PD activity and pigment production as markers of of exponential and stationary phase of growth, respectively. The antimicrobial activities are reported as % of growth inhibition (mm of inhibition halo on plates) considering 100% the maximal activity observed. In (**b**) the red arrow indicates the time of chloramphenicol supplementation. Grey bars indicate the % of antimicrobial activity (mm of inhibition halo on *L. monocytogenes* plates) considering 100% the maximal activity observed before the antibiotic treatment

*Bacillus pumilus* SF214 produces during its exponential phase of growth an antimicrobial molecule apparently bigger than 10 kDa active against the *Listeria monocytogenes* strain ATCC 7644 [8]. Production of antimicrobials during active growth is not common since most AMPs are secondary metabolites produced during the stationary phase of growth [3]. To confirm the previous observation and further characterize the synthesis of the anti-*Listeria* molecule, SF214 cells were grown in S7 medium at 25 °C, and samples collected at various times. For each time point, the cell-free supernatant was size-fractionated and the >10 kDa fraction tested for the anti-*Listeria* activity, as previously reported [8]. The cell extracts of all time points were assayed for activities known to occur during the exponential or stationary phase of growth: the glucose-6-phosphate dehydrogenase (G6PDH) and the production of the orange pigment, respectively [12, 11]. As reported in Fig. 1a, the anti-*Listeria* and G6PDH activities appeared during the active growth of SF214 cells (panel a) while the production of the pigment started later, at the entry into the stationary phase of growth (panel a). Results of Fig. 1a confirmed that actively growing cells of SF214 produce the anti-*Listeria* molecule but also indicated that the activity of such molecule appeared about one hour later than the G6PDH activity. This delay may be due to the secretion and eventual maturation/processing steps involved in the activation of some AMPs. An additional indication coming out of Fig. 1a is that the anti-*Listeria* activity persisted for several hours after its appearance.

This latter observation may be explained by a prolonged synthesis of the molecule, starting during the exponential growth and continuing during the stationary phase, or by the stability of the molecule that once produced remains active for a long time. To distinguish between these two possibilities, SF214 cells were grown as described above and the antibiotic chloramphenicol (5 µg/ml) added when cell reached 0.7 OD_600_ (red arrow in Fig. 1b), immediately blocking growth. Cell-free supernatants, collected right before as well as 1 and 3 h after the antibiotic treatment, were size-fractionated and >10 kDa fractions tested for the anti-*Listeria* activity in plate assays. An identical antimicrobial activity (mm of inhibition halo) was observed in the three samples, indicating that the inhibition of protein synthesis and growth did not reduce the antimicrobial activity and, therefore, suggesting that, once produced, the anti-*Listeria* molecule remains stable for at least three hours in the cytoplasm of resting cells (Fig. 1b).

Stability of the anti-*Listeria* molecule was also tested over a wide range of pH and temperature conditions and after treatments with chemicals and enzymes. The anti-*Listeria* molecule was similarly active against its target cells after 1 or 5 h of incubation at pH values ranging from 7.0 to 13.0, was only slightly reduced at pH 2.0 and 4.0 (Table 1). It was fully active after 15 min of incubation at 60 or 80 °C (Table 2) and slightly reduced after 15 min at 100 °C (Table 2). None of the organic solvents tested showed any effect on the antimicrobial that was totally degraded by both a trypsin or a proteinase K treatment (Table 2). Altogether, results of Tables 1 and 2 indicate that the anti-*Listeria* molecule is highly stable and of proteinaceous nature.

**Table 1:** Effect of pH on the anti-*Listeria* activity

pH	1 hour^a^	5 hours^a^
2	7.5	7.5
4	7.5	7.5
7	10	10
10	10	10
13	10	10

^a^ Diameter (mm) of the inhibition halo in plate assay

**Table 2:** Effect of enzymes, solvents and heat on the anti-*Listeria* activity

Treatment	Activity on plate assay^a^
None	10
Trypsin^b^	0
Proteinase K^b^	0
DNase^b^	10
Ribonuclease A^b^	10
Acetone^c^	10
Ethyl alcohol^c^	10
Chloroform^c^	10
Toluene^c^	10
15 min 60 °C	10
15 min 80 °C	10
15 min 100 °C	8

^a^ Diameter (mm) of the inhibition halo in plate assay

^b^ Enzyme concentration was 100 µg/ml

^c^ A 10% (v/v) concentration was used

### The anti-***Listeria*** molecule of SF214 is specifically active against ***L. monocytogenes***

The anti-*Listeria* molecule of SF214 has been previously found inactive against all other the Gram positive (*Streptococcus faecalis*, *Staphylococcus aureus, Bacillus megaterium*, *Mycobacterium smegmatis*, *Enterococcus faecalis*) or negative (*Citrobacter freundii*, *Pseudomonas fluorescens, Shigella sonnei, Escherichia coli, Salmonella enterica*) bacteria tested [8]. Here, the >10 kDa fraction of the SF214 cell-free supernatant was tested and found active against five other strains *L. monocytogenes* and but inactive against of *Listeria innocua* (Table 3). *L. innocua* is a non-pathogenic species of the *Listeria* genus, very similar to *L. monocytogenes* [14]. The two species have a genome of very similar size (2.94 and 3.01 Mb for *L. monocytogenes* and *L. innocua*, respectively), characterized by a high number of genes coding for surface and secreted proteins, transporters, and transcriptional regulators [15]. Although the human pathogen *L. monocytogenes* and the non-pathogenic *L. innocua* are strict relatives [14, 15], a comparative proteome analysis of the secreted and cell wall-associated proteins of the two bacteria revealed over 50 *L. monocytogenes-*specific proteins [16]. In addition to known virulence factors, this subgroup of proteins includes proteins with a putative role in cell wall metabolism, transporters, penicillin binding proteins, cell division and motility proteins [15]. The anti-*Listeria* molecule of SF214 is, therefore, highly specific and for this reason peculiar with respect to other AMPs that have a broad spectrum of action [1–3]. Examples are the recently discovered Amyloliquecidic GF610 of *B. velenzensis* [17] and a molecule produced by *B. amyliquefaciens* JFL21 [18], both active against *Listeria* but also against several Gram-positives and the latter also against some fungal pathogens. For its specificity and its proteinaceous nature the SF214 molecule will be therein indicate as specific anti-*Listeria* *m* *onocytogenes* peptide (SAMP) and all further analyses performed with *L. monocytogenes* ATCC 7644 as the target bacterium.

**Table 3:** List of Listeria strains used for the antimicrobial activity

Species	Strain
*Listeria monocytogenes*	ATCC 7644
*Listeria monocytogenes*	ATCC 19,115
*Listeria monocytogenes*	LM012018^a^
*Listeria monocytogenes*	LM001^b^
*Listeria monocytogenes*	ATCC 13,932
*Listeria monocytogenes*	ATCC 19,111
*Listeria innocua*	ATCC 33,090
*Listeria innocua*	BUG 499 [13] ^c^
*Listeria innocua*	DPC 1770^d^

Generous gifts from ^a^M. Guida (Federico II University, Naples, Italy); ^b^E. Ghelardi (University of Pisa, Pisa, Italy); ^c^B. Dupuy (Inst. Pasteur, Paris, France) ^d^D. Ercolini (Federico II University, Naples, Italy)

### SAMP has a lytic activity

**Fig. 2:**
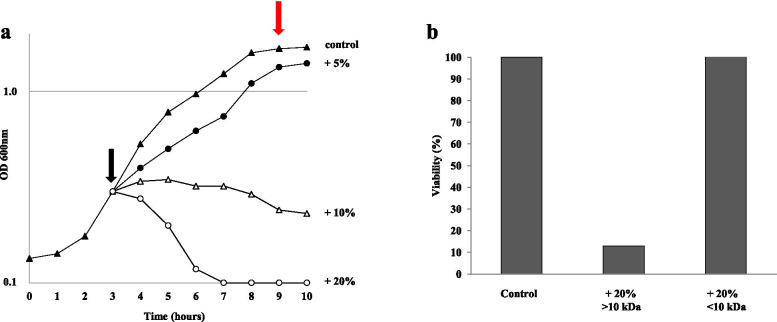
Antimicrobial activity of the supernatant (>10 kDa fraction of the cell-free supernatant) of SF214 on *L. monocytogenes* cells. **(a)** Different amounts of supernatant were added during growth at the time indicated by the black arrow. The red arrow indicates the time of collection of cells to test Antimicrobial activity on resting cells (see text). **(b)** MTT assay performed on *L. monocytogenes* cell without any supplementation (Control) or supplemented with the >10 or <10 kDa fractions of the SF214 cell-free supernatant

In order to characterize the antimicrobial activity of SAMP, *L. monocytogenes* cells were grown in LB medium at 37 °C, up to 0.3 OD_600_ (black arrow in Fig. 2a), then supplemented with different amounts of the >10 kDa fraction of the cell-free supernatant of SF214 and growth monitored for several hours. While 5% and 10% (vol/vol) of supernatant showed respectively a slight reduction of growth and a bacteriostatic effect on *L. monocytogenes*, 20% of it rapidly killed all cells, suggesting for the antimicrobial a lytic activity (Fig. 2a). Stationary cells of *L. monocytogenes* not treated with the anti-microbial were collected at the time indicated by the red arrow in Fig. 2a, incubated with supernatant 20% (vol/vol) for 3 and 16 h and plated for CFU analysis. No CFU reduction was observed (not treated: T_0_=1.10 × 10^9^; T_3_= 0.99 × 10^9^; T_16_=1.28 × 10^9^ CFUs; after supernatant treatment: T_0_=1.10 × 10^9^; T_3_=1.30 × 10^9^; T_16_=1.29 × 10^9^ CFUs), indicating that the antimicrobial was only active against growing cells of *L. monocytogenes.*

The lytic activity of SAMP was also confirmed by a colorimetric (MTT) assay that, by measuring the intracellular NAD(P)H-dependent oxidoreductase activity, indicates the metabolic activity of cells. As shown in Fig. 2b, growing cell of *L. monocytogenes* (OD600=0.6) treated with 20% (vol/vol) of the >10 kDa fraction of the SF214 cell-free supernatant, showed a strong reduction of the enzymatic activity and therefore of their viability with respect to the untreated cells and to cells treated with the same amount of the <10 kDa fraction of the SF214 cell-free supernatant, inactive against *L. monocytogenes*.

**Fig. 3:**
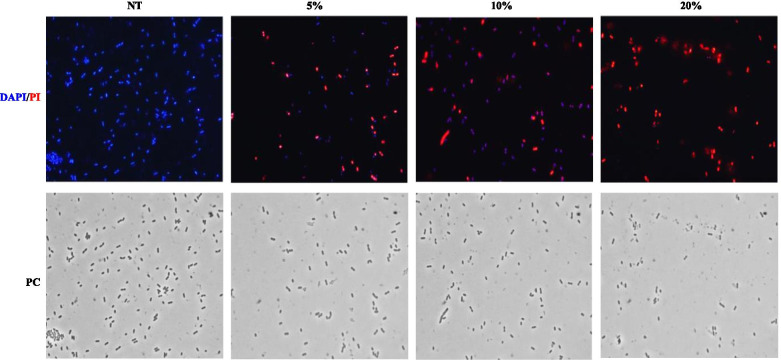
Viability assay by fluorescence and phase-contrast (PC) microscopy of *L. monocytogenes* cells stained with DAPI and PI. Cells were not treated (NT) or treated with increasing concentration of supernatant (from 5 to 20% vol/vol)

The effects of SAMP on *L. monocytogenes* cells were visualized by fluorescence microscopy after co-staining of the cells with 4,6-diamidino-2-phenylindole (DAPI) and propidium iodide (PI). The membrane-permeable DAPI stains all cells that appear blue while PI enters only damaged/death cells that appear red [19, 20]. As reported in Fig. 3, only blue cells were observed when the supernatant was not used while death (red) cells appeared after the antimicrobial treatment. Incubation for 4 h with increasing concentrations of the antimicrobial produced an increasing number of damaged/death (red) cells and only red cells were found at the highest concentration of antimicrobial used in the experiment (20%) (Fig. 3). This assay clearly shows an increased permeability of the *L. monocytogenes* cells in response to the treatment, indicating that the bactericidal effect occurs through membrane permeabilization and surface damages.

**Fig. 4:**
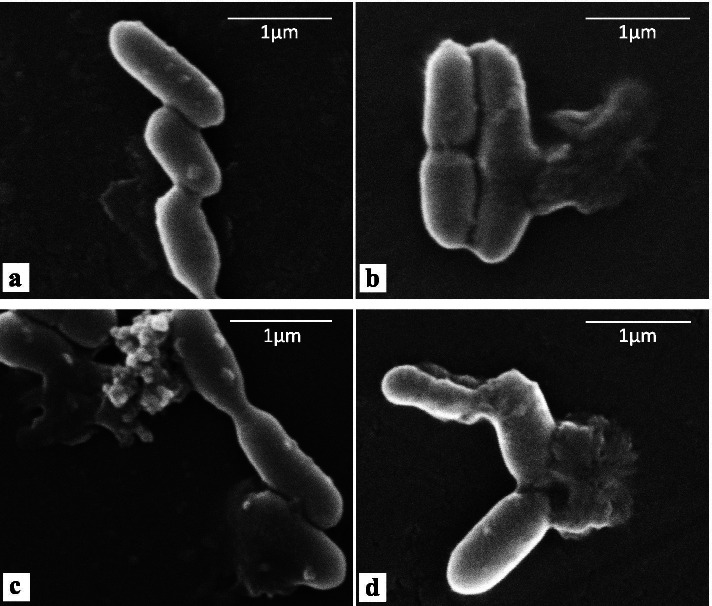
Scanning Electron Microscopy (SEM) analysis of *L. monocytogenes* cells not treated (**a**) or treated for 4 h with 20% (vol/vol) of supernatant (**b,c,d**). White arrows indicate points of cell rupture

The morphological defects due to the supernatant were visualized by a Scanning Electron Microscopy (SEM) analysis. *L. monocytogenes* cells treated for 4 h with 20% (vol/vol) supernatant showed distinct morphological changes (Fig. 4). Untreated cells appeared intact (Fig. 4a) while supernatant-treated cells showed on their surface cell debris (white arrows in Fig. 4b, c, d), an indication of the loss of cell integrity, similar to what detected with other antimicrobials affecting the membrane integrity [20, 21]. Interestingly, the defects observed after treatment with SAMP appeared different from those caused by another anti-*Listeria* compound. After a treatment with the commercially available Linalool the *Listeria* cell surface appeared by SEM analysis as a wrinkled [22] and did not show the cell debris observed in Fig. 4, suggesting a different mechanism of action for Linalool and SAMP.

To evaluate the toxicity of SAMP against eukaryotic cells, an MTT assay with human epithelial (Caco-2) cells was performed. SF214 supernatant w (20% vol/vol) was added to the growth medium of the Caco-2 cells. No effects on cell viability were observed after 24 h of incubation suggesting that SAMP is not toxic for human cells (not shown).

### Characterization of SAMP

**Fig. 5:**
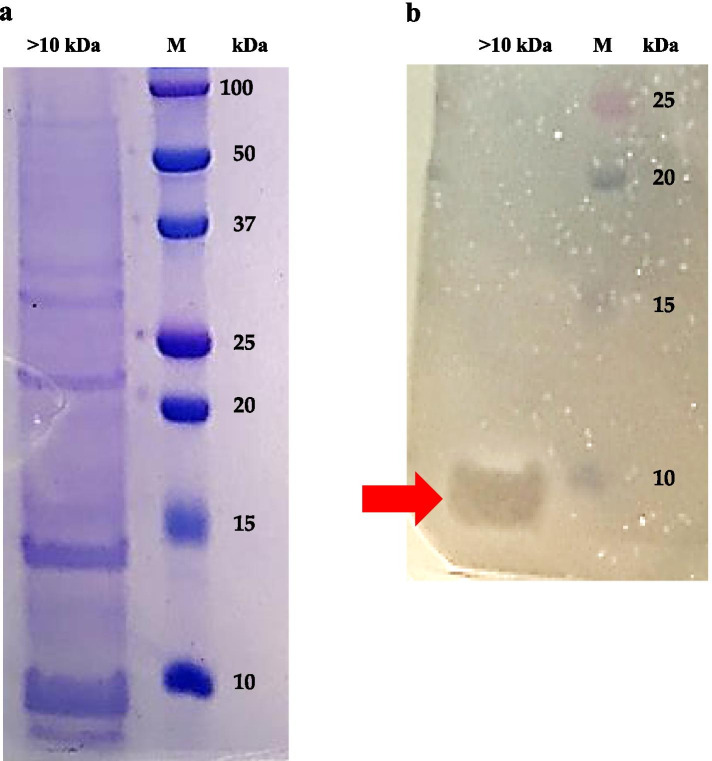
SDS-PAGE analysis of the >10 kDa fraction of the cell-free supernatant of SF214. (**a**) Coomassie-Blue stained 18% polyacrylamide gel. (**b**) 18% polyacrylamide gel not stained but fixed and overlaid with *L. monocytogenes* cells. The red arrow points to the inhibition halo observed after 18 h of incubation at 37 °C. M: molecular marker

To expand the preliminary observation that SAMP has a proteinaceous nature (Tables 1 and 2), the >10 Da fraction of the SF214 supernatant was analyzed on a 18% SDS-PAGE. As shown in Fig. 5a, about a dozen proteins ranging from slightly less than 10 to over 50 kDa was observed. A second gel was run in parallel, not stained with Coomassie-Blue but fixed and overlaid with soft-agar containing *L. monocytogenes* cells for direct detection of antimicrobial activity as previously described [23]. As shown in Fig. 5b, the anti-*Listeria* activity was associated with a band of apparent molecular mass slightly smaller than 10 kDa.

Although smaller than 10 kDa (Fig. 5b), SAMP did not pass through filters with a 10 kDa cut-off, suggesting the formation of aggregates dissolved during the SDS PAGE. To evaluate whether such potential high molecular weight aggregates were due to hydrophobic interactions, different amounts of methanol were added to the cell-free supernatant of SF214 to obtain final methanol concentrations of 20, 50 or 75%. Methanol was selected as solvent because it is known to weaken the hydrophobic interactions between proteins or between amino acids of the same protein [24] and because it did not have any inhibitory activity on *L. monocytogenes* at the used concentrations (not shown). After size-fractionation the <10 and >10 kDa fractions of the supernatant were diluted to the same final volume (10 ml) and aliquots tested for anti-*Listeria* activity. While 20% methanol did not have effects on SAMP and the anti-*Listeria* activity was found only in the >10 kDa fraction, in the presence of 50 and 75% methanol the activity was found only in the <10 kDa fraction (Table 4), suggesting that in 50 and 75% methanol SAMP did not form aggregates as in water or 20% methanol. Formation of high molecular weight aggregates and micelles by antimicrobials produced by *Bacillus* has been recently reported [25], supporting the conclusion based on data of Fig. 5; Table 4.

**Table 4:** Effect of methanol on antimicrobial activity

Methanol (%)	>10 kDa^a^	<10 kDa^a^
0	+	-
20	+	-
50	-	+
75	-	+

^a^ Inhibition halo in plate assay: - = no halo; + =presence of halo

#### Genome analysis

The genome sequence of SF214 [11] was analysed to search for homologs of genes coding for potential antimicrobial peptides and for NRPS/PKS, responsible for the non-ribosomal synthesis of peptides and polyketides. The coding gene of most ribosomally-synthesized antimicrobials produced by members of the *Bacillus* genus has not been identified, therefore the search for homologs was limited to some of the AMPs whose coding gene is known. As reported in Table 5, the SF214 genome encodes proteins with only a limited identity with known antimicrobials produced by Bacilli. The highest identity observed was 53% found between WP_060596419.1 of SF214 and Subtilosin A of *B. subtilis* (Table 5). However, Subtilosin A unlikely corresponds to SAMP since it is active against members of the *Bacillus* and *Enterococcus* genus [26] that are not affected by SAMP [8]. A bioinformatic analysis, performed by using the Anti-Smash (https://antismash.secondarymetabolites.org) and tools available on the PKS/NRPS Analysis Website (http://nrps.igs.umaryland.edu/), identified four clusters potentially coding for NRPS/PKS. These four clusters were further analysed by the HMMER tool (https://www.ebi.ac.uk/Tools/hmmer/) [27] to predict their potential products (Table 5). Cluster SF214_0323-SF214_0328 corresponds to the *srfA-sfp* locus, responsible of the synthesis of the Pumilacidin active against *S. aureus* and previously characteried [8], while the other three clusters were predicted to encode products formed by one (SF214_0601), two (SF214_3715-SF214_3717) or six (SF214_0611-SF21_0615) amino acids (Table 6), all too small for the predicted size of SAMP. Therefore, we conclude that none of the clusters identified by our *in silico* analysis is likely to encode for the amino acids forming SAMP. Based on the results of Tables 5 and 6, we hypothesize that SAMP is produced by transcription and translation of a structural gene and that is not a previously characterized molecule. Confirming such hypothesis will be a future challenge that will need to be based on the chemical characterization of the antimicrobial, a step so far impaired by the small amount of SAMP produced by SF214.

**Table 5:** Percentage of identity of known ribosomally-synthesized antimicrobials of Bacilli with proteins encoded by the SF214 genome

Antimicrobials	Highest Identity (%) hit on the SF214 translated genome
Subtilin [27]	WP_060597202.1 (37)
Bacthuricin F4 [28]	WP_060595519.1 (31)
Subtilin JS-4 [29]	WP_060597425.1 (34)
Cerecin [28]	WP_060597533.1 (39)
Antimicrobial peptide LCI [30]	WP_060596412.1 (47)
EricinA [27]	WP_060597202.1 (36)
Mersacidin [27]	WP_060596204.1 (34)
EricinS [27]	WP_012010311.1 (45)
Sublancin 168 [27]	WP_050944681.1 (42)
Subtilosin A [27]	WP_060596419.1 (53)
Clausin [30]	WP_060595627.1 (42)
Lichenicidin [30]	WP_003217179.1 (43)
Plantazolicin [30]	WP_060596638.1 (38)
Haloduracin [31]	WP_060595537.1 (25)
Amyloliquecidin GF610 [17]	WP_034659961.1 (43)
Coagulin A [28]	WP_060596970.1 (31)

**Table 6:** Genes potentially coding for NRPS/PKS^a^ in the SF214 genome

Cluster	Predicted product
SF214_0323-SF214_0328	glu-leu-leu-X-asp-leu-ile-val
SF21_0601	asn
SF214_0611-SF214_0615	asn-ohmal-glu-ohmal-ohmal-ohmal^b^
SF214_3715-SF214_3717	gly-tyr

^a^ NRPS: non-ribosomal peptide synthase, PKS: polyketide synthase;

^b^ ohmal: hydroxymalonil

## Conclusions

The main conclusion of this report is that *B. pumilus* SF214 during its exponential phase of growth produces an antimicrobial with a lytic activity specifically against growing cells of *L. monocytogenes*. Production of an antimicrobial by a *Bacillus* strain is clearly not a new observation since hundreds of them have been previously described. However, SAMP has unravelled some peculiar properties that make this molecule unusual and worth studying:

- SAMP is not a typical secondary metabolite, as most AMPs [2, 3, 6, 7]. It is produced at the mid exponential phase of growth of *B. pumilus* SF214 but about one hour later than G6PDH. Such delay could be due to the need of post-synthesis maturation events (for example, processing of a secretion signal), inducing the hypothesis that it is a ribosomally synthesized peptide. This hypothesis is also supported by the *in-silico* analysis of the SF214 genome, that did not show the presence of homologs of already known AMPs or of genes coding for NRPs suited to synthesize the anti-*L. monocytogenes* molecule.
- SAMP affects cell wall integrity of growing but not resting cells of *L. monocytogenes*, suggesting the cell wall synthesis machinery rather than structural components of the cell membrane as the target of action.
- SAMP is specifically active against *L. monocytogenes* and is not active even against strains of the close relative species *L. innocua*.
- SAMP is not toxic for human cells.

## Methods

### Bacterial strains, growth conditions and preparation of cell-free fractions

*Bacillus pumilus* SF214, *Listeria monocytogens* and *L. innucua* strains reported in Table 3 were grown either in LB broth (8 g/L NaCl, 10 g/L tryptone, 5 g/L yeast extract), BHI (Brain Heart Infusion) or (only *B. pumilus*) in S7 minimal medium (50 mM morpholine-propane-sulfonic acid (MOPS) (adjusted to pH 7.0 with KOH), 10 mM (NH_4_)_2_SO_4_, 5 mM potassium phosphate (pH 7.0), 2 mM MgCl_2_, 0.9 mM CaCl_2_, 50 µM MnCl_2_, 5 µM FeCl_3_, 10 µM ZnCl_2_, 2 µM thiamine hydrochloride, 20 mM sodium glutamate, 1% glucose, 0.1 mg/mL phenylalanine, and 0.1 mg/mL tryptophan) and cells grown in aerobic conditions at 25 °C. SF214 cultures were centrifuged (1000× g for 10 min at Room Temperature) and the supernatant filter-sterilized with a 0.22-µm filter (Millipore, Bedford, MA, USA). The supernatants were size-fractionated (10-kDa cutoff spin column; Centricon, Millipore) by centrifuging 11,000 g for 10 min at 4 °C. The fraction <10 kDa was concentrated 5 folds by a vacuum speed concentrator (Eppendorf).

### Antimicrobial activity on Plate Assay and on gel

Antimicrobial activity was determined as previously described [23] with the following modifications: 10 µL of concentrated (see above) <10 or >10 kDa fractions of the cell-free supernatant were spotted on the surface of a sterile LB agar plate and the spots air-dried. 100 µL of *L. monocytogenes* or *L. innocua* culture was mixed with 10 mL of soft agar (0.7%) and poured over the plate. Fresh media and ampicillin (1 µg/mL) were used as negative and positive controls, respectively. The plates were incubated aerobically overnight at 37 °C and the inhibition halos were measured and reported in mm.

For the direct detection of the antibacterial activity on gel, 50 ug of total proteins of the >10 Da fraction of SF214 cell-free supernatant were split in two identical samples and loaded in two independent lanes of a sodium dodecyl sulfate-polyacrylamide gel electrophoresis (SDS PAGE, 18%). After about 1.5 h, the gel was removed and cut into two vertical parts. One part of the gel, containing one sample and the molecular weight standards, was stained with Coomassie brilliant blue R250 (Sigma). The other part of the gel was tested for antimicrobial activity as previously described [23] with the following modifications: the gel was fixed immediately by a 2-h treatment in 20% isopropanol-10 mM Tris-HCl (pH 7.5) and 1-hour treatment in 20% isopropanol (pH 7.5) and 10% acetic acid, and at the end washed for 3 h in 10 mM Tris-HCl (pH 7.5). The gel was then placed into a petri dish, air-dried for 10 min and overlaid with 15 ml of 0.7% agar containing 10^6^ cells of the indicator strain. The dish was then incubated at 30 °C for about 16 h and analyzed for an inhibition halo.

### Detection of pigment production and of G6PDH activity

To evaluate pigment production, SF214 growth cultures were centrifugated at 7000 rpm for 10 min, the pellet was washed two times with lysis buffer (50 mM Tris-HCl pH 7.5, 1 mM DTT, 0.1 mM PMSF,10% glycerol), suspended in the same buffer and sonicated at 4 °C for 10 min (30 s. ON and 30 s. OFF). After centrifugation at 13,000 rpm for 15 min, supernatants were used to quantify the total protein concentration by the Bradford assay using bovine serum albumin (BSA) as standard protein. Samples of identical protein concentration were then used to determine the pigment content by following the adsorbance spectrum between 300 and 550 nm, as previously reported [10].

G6PDH activity was analyzed at 25 °C by measuring the reduction of NADP+ to NADPH at 340 nm by G6PDH in the presence of glucose-6-phosphate (G6P) in a 1 cm cuvette in a spectrophotometer Cary 60 (Agilent Technologies) as previously reported [12]. Assays were always carried out in duplicates. The reaction mixture (final volume 1 ml) contained 5 mM MgCl_2_, 150 µM NADP+, and 3 mM G6P in 30 mM Tris–HCl buffer, pH 7.5. 50/100µl of supernatant were utilized; blank without G6P. One unit of enzyme (U) activity defined as the amount of enzyme that reduced 1 µmol NADP+ per minute, the total activity was expressed as units per mg of protein. The determination of total protein concentrations was performed as described above.

### Stability of Antimicrobial at Different pH, Temperature, Chemical, and Enzyme Conditions

The effects of pH and heat on supernatants were analyzed by assaying the antimicrobial activity after 1 or 5 h of incubations at 30 °C in 50 mM phosphate buffer (pH 6.0), adjusted to the various pH with HCl and NaOH and after 15 min of incubation at 60, 80, and 100 °C (Tables 1 and 2). Enzymes (100 µg/mL) and 10% organic solvents were added to 100 µL of culture supernatant. Enzyme-treated samples were incubated 1 h at 37 °C (42 °C in the case of proteinase K) and solvent-treated samples were incubated for 1 or 5 h at 25 °C, and subsequently, 10 µL aliquots were tested for antimicrobial activity as described above (Table 2).

### MTT assays

Cytotoxicity on *L. monocytogenes* cells was assessed by performing the (3-(4,5-dimethylthiazol-2-yl)-2,5 diphenyltetrazolium bromide) (MTT) reduction inhibition assay. Cells were grown in LB medium at 37 °C, thoroughly washed and resuspended in PBS + 0.2% glucose reaching a final OD_600_ of 0.6. 100ul of cells were incubated at 37 °C with SF214 cell-free supernatant >10 kDa 20% (v/v), SF214 cell-free supernatant <10 kDa 20% (v/v) and PBS 0.2% glucose (control) respectively. After 3 h of incubation cells were gently centrifuged to remove the supernatant, resuspended in 90ul of PBS + 0.2% glucose, moved into 96-well plate and 10 µl of a stock MTT reagent was added into each well. The samples were incubated on a thermostatic shaker at 37 °C and 200 rpm in the dark. After 30 min, 100 µl of DMSO per well was added and plates were incubated for 15 min at room temperature in the dark (complete formazan dissolution was detected). The absorbance of each well at 560 nm was measured using a microplate reader (Thermo, Multiskan Spectrum) [32]. The MTT reagent was prepared by dissolving 5 mg of 3-(4,5-dimethylthiazol-2-yl)-2,5-diphenyl-tetrazolium bromide (MTT) (Sigma-Aldrich) in 1 ml PBS. DMSO was used for solubilization of the formazan crystals.

Cytotoxicity on human cells was performed by using epithelial (Caco2) cells that were cultured in Dulbecco’s modified Eagle’s medium (Sigma Aldrich, Milan, Italy) supplemented with 10% fetal bovine serum (HyClone, GE Healthcare Lifescience, Chicago, IL) and 1% penicillin-streptomycin, at 37 °C in humidified atmosphere of 5% CO2.

Cytotoxicity on CaCo-2 cells was assessed by performing the MTT reduction inhibition assay. Cells were grown as described and plated on 96 well plates at a density of 5 × 10^3^ cells per well, in 200 µl of medium containing the >10 kDa fraction of the SF214 cell-free supernatant (20% v/v) for 24 h. After treatment, the medium was eliminated and 10 µl of a MTT solution (5 mg/ml) was added to the cells to a final concentration of 0.5 mg/ml. After 4 h incubation, the MTT solution was removed and the MTT formazan salts were dissolved in 100 µl of DMSO. Cell survival was expressed as the absorbance of blue formazan measured at 570 nm with an automatic plate reader (Multi scan spectrum; Thermo-Fisher Scientific, Waltham, MA, USA). Cytotoxicity tests were performed at least 3 times and cell survival values expressed as percentage of viable cells with respect to control untreated sample.

### DAPI/IP dual staining, fluorescence and SEM microscopy

For dual staining, 200 µl of bacterial culture (grown to mid-logarithmic phase) were incubated in the dark for 4 hours at 37°C in agitation without or with increasing concentration (5 to 20% vol/vol) of the >10 kDa fraction of the SF214 cell-free supernatant. After the incubation 10 µl of bacterial culture were mixed with 1.5 µl DAPI solution (4’,6-diamidino-2-phenylindole dihydrochloride; Sigma Aldrich) (1 µg/ml DAPI final concentration) and 1.5 µl of PI (propidium iodide 20 µg/ml final concentration; Sigma Aldrich) and incubated in the dark for 30 min at room temperature [19]. Samples were observed with an Olympus BX51 fluorescence microscope (Olympus, Tokyo, Japan) using a DAPI/TRITC filters *(s*tandard acquisition times were 200 ms). Images were captured using an Olympus DP70 digital camera.

For SEM analysis, *L. monocytogenes* cells were grown in LB to an exponential phase, harvested by centrifugation at 11,000 g for 10 min and the pellet resuspended in 10 ml of phosphate buffer (pH 7.0). An aliquot of 5 ml containing 1 × 10^8^ cells/ml was incubated at 37 °C for 4 h with 20% SF214-CFS (v/v) while the control (untreated) sample was incubated with phosphate buffer at the identical conditions. Then the samples were cut from subapical parts using a sharp razor blade, fixed with 3% glutaraldehyde in phosphate buffer (65 mM, pH 7.2–7.4) for 2 h at room temperature, post-fixed with 1% osmium tetroxide in the same phosphate buffer for 1.5 h at room temperature, and completely dehydrated with ethanol and critical point drying. Both samples were then mounted on aluminum stubs, coated with a thin gold film using an EdwardE306 Evaporator, and observed with a FEI (Hillsboro, OR, USA) Quanta 200 ESEM in high vacuum mode (P 70 Pa, HV 30 kV, WD10 mm, spot 3.0).

## Data Availability

All data generated or analyzed during this study are included in this published article. Raw data are available on request by contacting the corresponding author by email (ericca@unina.it).

## Abbreviations

AMP: Anti-Microbial Peptide
FA: Fatty acid
NRPS: Non-Ribosomal Peptide Synthase
G6PDH: Glucose-6-Phosphate DeHydrogenase
SAMP: Specific Anti-*Listeria Monocytogenes* Peptide
CFU: Colony Forming Units.
MTT: 3-(4,5-dimethylthiazol-2-yl)-2,5 diphenyltetrazolium bromide
IP: propidium iodide.
DAPI: 4’,6-diamidino-2-phenylindole dihydrochloride.
SEM: Scanning Electron Microscopy.
